# Pectin-rich biomass as feedstock for fuel ethanol production

**DOI:** 10.1007/s00253-012-4173-2

**Published:** 2012-06-14

**Authors:** Meredith C. Edwards, Joy Doran-Peterson

**Affiliations:** Department of Microbiology, University of Georgia, 1000 Cedar Street, 550, Biological Sciences, Athens, GA 30602 USA

**Keywords:** Pectin, Ethanol, Biofuels, Fermentation

## Abstract

The USA has proposed that 30 % of liquid transportation fuel be produced from renewable resources by 2030 (Perlack and Stokes 2011). It will be impossible to reach this goal using corn kernel-based ethanol alone. Pectin-rich biomass, an under-utilized waste product of the sugar and juice industry, can augment US ethanol supplies by capitalizing on this already established feedstock. Currently, pectin-rich biomass is sold (at low value) as animal feed. This review focuses on the three most studied types of pectin-rich biomass: sugar beet pulp, citrus waste and apple pomace. Fermentations of these materials have been conducted with a variety of ethanologens, including yeasts and bacteria. *Escherichia coli* can ferment a wide range of sugars including galacturonic acid, the primary component of pectin. However, the mixed acid metabolism of *E. coli* can produce unwanted side products. *Saccharomyces cerevisiae* cannot naturally ferment galacturonic acid nor pentose sugars but has a homoethanol pathway. *Erwinia chrysanthemi* is capable of degrading many of the cell wall components of pectin-rich materials, including pectin. *Klebsiella oxytoca* can metabolize a diverse array of sugars including cellobiose, one degradation product of cellulose. However, both *E. chrysanthemi* and *K. oxytoca* produce side products during fermentation, similar to *E. coli*. Using pectin-rich residues from industrial processes is beneficial because the material is already collected and partially pretreated to facilitate enzymatic deconstruction of the plant cell walls. Using biomass already produced for other purposes is an attractive practice because fewer greenhouse gases (GHG) will be anticipated from land-use changes.

## Introduction

The search for a fossil fuel alternative has become increasingly important in the USA due to many factors including: the finite availability of fossil fuels, strained foreign relations between the USA and petroleum providers, and the negative effect burning fossil fuels has on the environment. The USA currently consumes approximately 20 million barrels of crude oil every day. Of these 20 million barrels, over 60 % is imported (US Energy Information Administration, www.eia.doe.gov). Since almost 70 % of the crude oil is used for transportation fuels, an increase in alternative and renewable transportation fuels is vital to reduce the total amount of imported crude oil consumed in the USA.

There are many proposed methods to alleviate the USA’s dependence on petroleum-based fuels. One notable alternative is ethanol based biofuels produced from plant biomass. Currently, in the USA, ethanol is primarily produced from starch in corn kernels. However, corn kernels alone will not produce enough ethanol to meet the goals set forth in The Billion Ton Report which proposed that 30 % of liquid transportation fuels should be produced from renewable resources by 2030 (Perlack and Stokes 2011). Therefore, it is necessary to consider other biomass sources for the production of ethanol in congruence with corn kernel ethanol production.

The USA is capable of producing between 1 to 1.6 billion dry tons of biomass per year, which could provide enough ethanol to displace almost 30 % of current crude oil usage (Perlack and Stokes 2011). Using biomass that is a by-product or process residue is an attractive practice because fewer GHG will be produced from land-use change. Currently, lignocellulosics, including residues from existing biomass processing, are being vigorously investigated to augment corn kernel ethanol production.

Lignocellulosic biomass is much more complex than corn kernels and is composed of 25–55 % cellulose, 24–50 % hemicellulose, and 10–35 % lignin on a dry weight (dw; Pettersen 1984; Dale et al. 1996; Sun and Cheng 2002). Lignin decreases enzymatic degradation of the plant cell wall polysaccharides (Chang and Holtzapple 2000; Berlin et al. 2005; Guo et al. 2009). Pectin-rich biomass has a low lignin concentration and increased pectin concentration, ranging from 12 % to 35 % of the biomass dw (Kennedy et al. 1999; Doran et al. 2000; Mohnen 2008; Zhou et al. 2008). Pectin-rich biomass is an abundant and widely underused resource and includes residues such as apple pomace, citrus waste, and sugar beet pulp (Table 1). All of these biomass types are waste residues left after the fruit or vegetables have been processed for juice or sugar production.

**Table 1:** Production and waste generation from pectin-rich biomass; apple, citrus, and sugar beet in the United States in millions of tons. Possible ethanol generation from these wastes in million tons. Production tons are 2009 data from http://www.faostat.far.org

	(wet wt)		(dry wt)		Source
	Production	Waste	Waste	Ethanol	
Apple	4.5	1.5	0.4	0.08	(Chong 1992; Kennedy et al. 1999)
Citrus	10.7	4.6	0.8	0.30	(Braddock 1995; Zhou et al. 2008)
Sugar beet	26.8	5.4	1.6	0.62	(Doran et al. 2000)
Total	43.2	12.7	2.8	1.00	

Cost estimates for ethanol production from citrus waste was modified from the cellulose-to-ethanol process model from NREL and USDA/ARS (Wooley et al. 1999; McAloon et al. 2000; Aden et al. 2002) and estimated to be $1.23/gal. While more expensive than corn kernel ethanol ($1.00/gal), citrus waste ethanol is predicted to be cheaper than lignocellulosic ethanol processes ($1.35–1.62/gal; Zhou et al. 2007). One reason a citrus waste-to-ethanol process may be more economically viable than lignocellulosic ethanol processes is the generation of the citrus-derived co-product limonene, which can be sold to help off-set ethanol production costs (Zhou et al. 2007).

## Pectin structure

A brief description of pectin is provided; however, for a more detailed review on pectin structure and biosynthesis see Mohnen (2008). Pectin is a complex carbohydrate primarily composed of covalently linked galacturonic acids (70 %). Pectin may also contain rhamnose, xylose, arabinose, and galactose (Mohnen 2008).

The three most prominent types of pectin present in the cell wall are homogalacturonan, rhamnogalacturonan I, and rhamnogalacturonan II. Homogalacturonan (an α-1,4-linked linear polymer of galacturonic acid) accounts for roughly 65 % of pectin (Mohnen 2008). The second most prominent type of pectin is rhamnogalacturonan I which comprises 20–35 % of pectin (Mohnen 2008). Rhamnogalacturonan I has a disaccharide backbone composed of galacturonic acid and rhanmose. The rhamnose molecules are highly substituted with a variety of side chains primarily composed of arabinans and galactans (Willats et al. 2001; Mohnen 2008). Rhamnogalacturonan II is composed of a homogalacturonan backbone substituted with 12 different sugars and comprises approximately 10 % of pectin in the cell wall (O’Neill et al. 2004; Mohnen 2008).

## Pectin-rich biomass composition

**Fig. 1:**
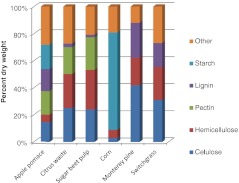
A comparison of the dry weight composition of pectin-rich biomass to starches and other lignocellulosic biomasses. Pectin-rich biomass includes citrus waste, apple pomace, and sugar beet pulp. (Apple pomace (Kennedy et al. 1999), citrus waste (Zhou et al. 2008), sugar beet pulp (Doran et al. 2000), corn kernels(Lynd et al. 1999), Monterey pine and switchgrass http://www.afdc.energy.gov/biomass/progs/search1.cgi)

The cell walls of pectin-rich biomass contain 12–35 % pectin on a dry weight (dw) basis (Kennedy et al. 1999; Doran et al. 2000; Mohnen 2008; Zhou et al. 2008). In comparison, cell walls of biomass that are not characterized as pectin-rich, such as corn kernels, grasses, and woody biomass, only contain 2-10 % dw pectin in their cell wall (Mohnen 2008). Figure 1 compares the composition of pectin-rich materials (apple pomace, citrus waste, and sugar beet pulp) to other biomass types (corn kernels, Monterey pine, and switchgrass). All of these contain a significant amount of cellulose except the corn kernel, which is predominantly starch.

Pectin-rich residues have notably less lignin than lignocellulosic biomass. Approximately 2 % dw of citrus waste and sugar beet pulp is lignin, much less than the 26 % dw of Monterey pine. This is significant because lignin interferes with the enzymatic degradation of cellulose and hemicellulose (Chang and Holtzapple 2000; Berlin et al. 2005; Guo et al. 2009) and is not fermentable into ethanol. Before lignocellulosic material can be fermented to produce ethanol, lignin bonds with carbohydrates must be broken. This often requires costly and harsh physical, chemical and/or biological pretreatments that may degrade lignin and some sugars into inhibitory molecules. A review of these pretreatments can be found in Kumar et al. (2009).

## Applications

An economical and environmentally sustainable use of waste products generated by the industrial processing of fruits has been sought for centuries. There are a variety of uses for the pectin-rich waste material. Some proposed uses of apple pomace include, use as an animal feed, fertilizer, insect bait, ion exchange resin, incorporation into human foods, production of wine, pectin, methane, ethanol, citric acid, butanol, enzymes, apple seed oil, apple vinegar, apple wax, aroma compounds, flavoring, oxalic acid, xyloglucan, activated carbon, antioxidants, heteropolysaccharides, and furfural (Kennedy et al. 1999; Bhushan et al. 2008; Vendruscolo et al. 2008). Citrus waste, sugar beet pulp, and other food wastes can be used in many similar applications (Hang 2006).

Currently the most common use of pectin-rich biomass is as animal feed. Pectin-rich biomass tends to be low in crude protein, fat and phosphorus but high in fiber content and calcium (Table 2). However, it has been shown to be a good feed supplement if added in the correct amounts. For example, citrus waste should compose less than 50 % of beef cattle diet; diets of greater than 60 % citrus waste can cause cattle to develop ruminal parakeratosis (Arthington et al. 2002).

**Table 2:** The recommended range (in percent dry matter) of crude protein (CP), fats, neutral detergent fiber (NDF), acid detergent fiber (ADF), calcium, and phosphorus in dairy cattle feed compared to actual quantities found in pectin-rich materials

	CP	FAT	NDF	ADF	Calcium	Phosphorus	Source
Recommended	10-19	3.5–5.0	23–35	0.16–0.25	0.16–0.25	0.65–1.00	(Brandt and Martin 1994)
Apple pomace	5.40	1.5–2.3	42.52	0.14	0.14	0.09	(Grohmann and Bothast 1994; Kennedy et al. 1999)
Citrus waste	6.40	0.7–1.5	20.35	1.58	1.58	0.12	(Grohmann and Bothast 1994; Arthington et al. 2002)
Sugar beet pulp	9.63	<0.1	63.36	0.97	0.97	0.06	(Grohmann and Bothast 1994)

Unfortunately, selling these waste products results in relatively nominal economic returns due to the high cost of dehydrating and pelletizing the material (Doran et al. 2000; Vendruscolo et al. 2008). Sugar beet pulp drying and pelleting can comprise up to 30 to 40 % of the overall energy cost of the waste processing (Coons 1982) and the selling price for these pectin-rich residues varies (Grohmann et al. 1998). The low economic value of pectin-rich materials as an animal feed source makes finding alternative applications for this biomass appealing. One possible alternative is ethanol production for biofuels.

## Ethanol production

The industrial processing of fruits resulting in pectin-rich waste produces a favorable biomass for ethanol production. First, the biomass is conveniently stockpiled in relatively large amounts at the processing facilities, significantly decreasing the cost of collection and transportation (Doran et al. 2000). Secondly, industrial processing can reduce the pretreatment required before fermentation is begun. Himmel et al. describes the complex process required for lignocellulosic degradation from the thermochemical pretreatment down to the enzymatic digestion to form monomeric sugars for ethanol production (Himmel et al. 2007). Highly recalcitrant biomass like grasses and woods require treatments such as ammonia fiber expansion (AFEX) or dilute sulfuric acid pretreatment (Kumar et al. 2009). Some pectin-rich biomass does require pretreatment to disrupt the biomass structure or to remove compounds inhibitory to fermentation, like limonene in citrus waste (von Loesecke 1934; Grohmann et al. 1994a, b; Wilkins et al. 2007b). However, sugar beet pulp fermentations do not require additional particle size reduction, chemical pretreatment, nor inhibitory compound removal. Most sugar beet pulp fermentations were conducted using autoclaving at 121 °C for 20 minutes to minimize contamination, followed by enzymatic digestion and fermentation of the resulting carbohydrates.

## Fermenting with *Saccharomyces cerevisiae*

Ethanol production data from pectin-rich biomass fermentations has been compiled based on biomass type in Table 3 (apple pomace), Table 4 (citrus waste), and Table 5 (sugar beet pulp) and some of these fermentations will be discussed in more detail. Early fermentations were conducted with *S. cerevisiae*. Some advantages of using *S. cerevisiae* include its ability to tolerate high substrate concentrations and high ethanol concentrations, as well as relatively low pH and oxygen levels (Gujjari et al. 2009), making it a robust organism for the fermentation process. It also converts sugars to ethanol using a homoethanol pathway; therefore, sugars are not siphoned into unwanted co-products. In fermentations where sucrose content is high, *S. cerevisiae* performs well. However, *S. cerevisiae* is not the ideal ethanologen for pectin-rich biomass pulp or residue fermentations, due to its inability to naturally ferment pentose sugars and galacturonic acid.

**Table 3:** A review of ethanol production from apple pomace fermentations using a variety of ethanologens with no additional commercial enzymes

Organism	Pretreatment	Ferm type	Solids^a^	Inoculum	pH	Temp (°C)	Max EtOH (%) ^b^	Time (h)	Reference
*S. cerevisiae* MTCC 173	none	solid state	100	1 % *v*/*w*	6.5	30	8.44	72	(Chatanta et al. 2008)
*S. cerevisiae*	rehydrated (1:4) with ammonium sulfate (1.8 %)	solid state	1000	5 % *v*/*w*	4.2-3.9	25	4.50	96	(Joshi and Sandhu 1996)
*S. cerevisiae* ATCC 24702	none	solid state	2500	1L^c^	n/a	30	2.08 *w*/*w*	40^d^	(Ngadi and Correia 1992)
*S. cerevisiae* Montrachet strain 522	none	solid state	800	25 ml (4 g dw)	n/a	30	4.30 *w*/*w*	24	(Hang et al. 1981)

^a^All solids loading are in g

^b^All maximum ethanol yields are in % *v*/*w* unless otherwise specified

^c^2 ml of stock culture was incubated at 30 °C for 3 days in 10 ml pressed apples, transferred to 1 l pressed apples and incubated at 30 °C for 3 days before inoculating fermentation

^d^These times are estimates from graphs

**Table 4:** A review of ethanol production from citrus waste fermentations using a variety of ethanologens

Organism	Pretreatment	Ferm type	Enzyme load	Solids	Inoculum	pH	Temp (°C)	Max EtOH (%)^a^	Time (h)	Reference
*S. cerevisiae*	live steam (150-160 °C/2-4 min)	SSF^b^	pectinase (60 IU/g dw), cellulase (15FPU/g dw), β-glucosidase (50 IU/g dw)	20 g	330 mg cells/l	5.0	35	1.34	24	(Peterson unpublished)
*E. coli* LY40A	live steam (150-160 °C/2-4 min)	PSCF^c^	pectinase (60 IU/g dw), cellulase (15FPU/g dw), β-glucosidase (50 IU/g dw)	20 g	330 mg cells/l	5.5	35	1.85	120	(Peterson unpublished)
*S. cerevisiae*	dilute sulfuric acid (0.8 % v/v pH 2.2) steam expansion (160 °C)	SSF^b^	pectinase (0.42 IU/g), cellulase (.066IFPU/g), β-glucosidase (0.594 IU/g)	100 g	1 mg cells/g	4.2	37	2.70	48	(Widmer et al. 2010)
*S. cerevisiae*	live steam (155 °C/410-550kPa/2 min)	SSF	Pectinase (60 IU/g dw), cellulase (0.035FPU/g dw), β-glucosidase (0.81 IU/g dw)	100 g	1 mg cells/g	4.9	37	3.48	48	(Widmer et al. 2009)
*K. marxianus*	live steam (155 °C/410-550kPa/2 min)	SSF	Pectinase (60 IU/g dw), cellulase (0.035FPU/g dw), β-glucosidase (0.81 IU/g dw)	100 g	1.26 mg cells/g	4.9	37	3.45	48	(Widmer et al. 2009)
*S. cerevisiae*	steam expansion (150 °C/70 psi)	SSF	pectinase, cellulase, β-glucosidase	n/a	n/a	4.2-4.8	38	4.05	18	(Zhou et al. 2008)
*S. cerevisiae*	live steam (150-160 °C/2-4 min)	SSF	pectinase (297 IU/g dw)	100 g	7 mg cells/g	5.0	37	3.96	24	(Wilkins et al. 2007b)
*E. chrysanthemi* EC16	ground, dilute sulfuric acid (0.06 % *v*/*v*, pH 2.0) autoclaved^e^	SF^d^ (pH 4.8)	pectinase, cellulase, β-glucosidase	10 %	0.2 g cells/L	7.0	30	1.28	n/a	(Grohmann et al. 1998)
*E. coli* KO11	ground	SF	pectinase (12.4U/g), cellulase(0.4IFPU/g), β-glucosidase (1.6 mg/g)	80 % *v*/*v*	330 mg cells/l	5.8	37	4.70	72	(Grohmann et al. 1995)
*E. coli* KO11	ground	SF	pectinase (12.4U/g), cellulase (0.37FPU/g), β-glucosidase (1.6 mg/g)	90 % *v*/*v*	0.24 g cells/l	5.8	37	2.76	72	(Grohmann et al. 1994b)
*S. cerevisiae*	ground	SF	pectinase (12.4U/g), cellulase(0.37FPU/g), β-glucosidase (1.6 mg/g)	22 % wt	10^8^ cells/ml	5.0	35	4.70	14	(Grohmann et al. 1994a)

^a^All maximum ethanol yields are in % *w*/*v* unless otherwise specified

^b^Simultaneous saccharification and fermentation

^c^Partial saccharification and cofermentation (partial saccharification conducted for 24 h at pH 4.5, 42 °C, unless otherwise specified

^d^Performed saccharification (24 h at pH 4.3-3.3, 45 °C, unless otherwise specified), then removed, filtered, and fermented hydrolysate

^e^All autoclaving was conducted at 121 °C/1 atm/20 min unless otherwise specified

**Table 5:** A review of ethanol production from sugar beet pulp fermentations using a variety of ethanologens. All biomass was pretreated by autoclaving (121 °C/1 atm/20 min) except *Clostridum thermocellum* which was autoclaved for 90 min

Organism	Ferm type	Enzyme load	Solids^a^	Inoculum^b^	pH	Temp (°C)	Max EtOH (%)^c^	Time (h)	Reference
*S. cerevisiae*	PSCF^d^	pectinase (170PGU/g dw), cellulase(5FPU/g dw)	10.0	330	5.0	35	1.74	24	(Peterson unpublished)
*E. coli* LY40A	PSCF	pectinase (170PGU/g dw), cellulase(5FPU/g dw)	10.0	330	5.0	35	2.77	72	(Peterson unpublished)
*E. coli* KO11	SSF^e^	pectinase (200ul/g dw)^f^, cellulase (3.75FP/g dw), cellobiase (7.5CBU/g dw)^g^	10.7 % *w*/*w* ^h^	1 % *v*/*v* ^i^	6.5	37	2.65	120	(Rorick et al. 2011)
*E. coli* KO11	SSF	pectinase (200ul/g dw)^f^, cellulase (3.75FP/g dw), cellobiase (7.5CBU/g dw)^gj^	10.7 % *w*/*w* ^h^	1 % *v*/*v* ^i^	6.5	37	1.98	120	(Rorick et al. 2011)
*S. cerevisiae* + *E. coli* KO11^k^	SSF	pectinase (200ul/g dw)^f^, cellulase (3.75FP/g dw), cellobiase (7.5CBU/g dw)^g^	10.7 % *w*/*w* ^h^	1 % *v*/*v* ^i^	5.0/6.5^l^	37	2.97	216	(Rorick et al. 2011)
*S. cerevisiae*	PSCF	pectinase (240.8PGU/g dw), cellulase (10.5FPU/g dw)	10.0	330	5.0	35	1.60	24	(Peterson 2006)
*E. coli* LY01	PSCF	pectinase (240.8PGU/g dw), cellulase(10.5FPU/g dw)	10.0	330	5.0	35	4.00	96	(Peterson 2006)
*K. oxytoca* P2	PSCF (pH 5.0)	pectinase (30 mg/g dw), cellulase (60/g dw)	10.6	330	5.5	35	2.10	96	(Sutton and Peterson 2001)
*E. coli* KO11	PSCF	pectinase (60.2 PGU/g dw), cellulase(5.25 FPU/g dw)	10.6	330	5.5	35	2.60	96^m^	(Doran et al. 2000)
*E. chrysanthemi* EC16 pLOI555	PSCF	pectinase (60.2 PGU/g dw), cellulase (5.25 FPU/g dw)	10.6	330	5.5	35	1.97	120^m^	(Doran et al. 2000)
*K. oxytoca* P2	PSCF	pectinase (60.2 PGU/g dw), cellulase (5.25 FPU/g dw)	10.6	330	5.5	35	2.11	96^m^	(Doran et al. 2000)
*E. coli* KO11	PSCF	pectinase (60.2 PGU/g dw), cellulase(5.25 FPU/g dw)	10.0	330	5.5	35	2.71	n/a	(Doran et al. 2000)
*E. coli* KO11	PSCF	pectinase (120.4 PGU/g dw), cellulase (10.5 FPU/g dw), β-glucosidase (6.4 CBU/g dw)	10.46 + 0.16 at 24 h	330	5.5	35	4.00	120^m^	(Doran et al. 2000)
*Clostridium thermocellum*	SSF	none	50	250 ml of 24 h subculture^i^	7.0	60	0.32	n/a	(Spinnler et al. 1986)

^a^All solids are in % *w*/*v* unless otherwise specified

^b^All inoculum levels are in mg dry wt cells/l unless otherwise specified

^c^All maximum ethanol yields are in % *w*/*v* unless otherwise specified

^d^Partial saccharification and cofermentation (partial saccharification conducted for 24 h at pH 4.5, 42 °C, unless otherwise specified)

^e^Simultaneous saccharification and cofermentation

^f^Pectinase activity was not presented, only volume added.

^g^FPU and CBU were calculated from data provided

^h^Solids cannot be converted to *w*/*v* with data provided

^i^Inoculum cannot be converted to dry wt of cells from data provided

^j^Pectinase was added at the beginning of the fermentation. After 4 days, cellulase and cellobiase were added to the fermentation

^k^Fermentations were first inoculated with *S. cerevisiae* at pH 5. After 3 days the pH was increased to 6.5 for a second fermentation with *E. coli* KO11

^l^ *S. cerevisiae* fermentation was conducted at pH 5, *E. coli* KO11 fermentation was conducted at pH 6.5

^m^These times are estimates from graphs

Strains of *S. cerevisiae* capable of fermenting xylose and arabinose have been developed. Xylose fermenting strains perform fairly well; however, arabinose fermenting strains still require optimization (Sedlak and Ho 2001; van Maris et al. 2006; Nevoigt 2008). Arabinose utilization is important when fermenting pectin-rich biomass due to the arabinans present on rhamnogalacturonan I (Mohnen 2008). In fact, arabinose comprises 18–21 % (dw) of sugar beet pulp (Renard and Thibault 1993; Micard et al. 1996). Engineering *S. cerevisiae* to utilize galacturonic acid has also been suggested and a general plan has been outlined (van Maris et al. 2006).

## Fermenting with *Escherichia coli*

Another option for pectin-rich biomass fermentations is *E. coli*. *E. coli* does not tolerate ethanol as well as *S. cerevisiae* and has a higher optimal pH (Gujjari et al. 2009). Therefore, *E. coli* and commercial enzymes required for degradation of the plant cell wall (which have an acidic optimum pH) cannot reach their maximum activities simultaneously during biomass fermentation. *E. coli* is capable of fermenting a wide range of sugars including arabinose and galacturonic acid. Galacturonic acid catabolism in *E. coli* has been reviewed previously (Richard and Hilditch 2009). To increase efficiency of fermentations using *E. coli*, strains have been bioengineered to produce higher titers of ethanol from biomass. Typically, when *E. coli* ferments sugars it produces mixed acids, including ethanol, acetate, formate, succinate and lactate (Conway et al. 1987; Dien et al. 2003; Jarboe et al. 2007). A strain of *E. coli*, KO11, was engineered to shunt pyruvate into a homoethanol producing pathway and away from *E. coli*’s native pathways (Ohta et al. 1991).

Fermentations of pectin-rich biomass conducted with KO11 produced higher ethanol titers than fermentations performed with *S. cerevisiae*. Citrus waste fermented by *S. cerevisiae* (pH 6, 37 °C) produced 3.96 % (*w*/*v*) ethanol (Wilkins et al. 2007b) but citrus waste fermented by *E. coli* KO11 (pH 5.8, 37 °C) produced 4.70 % (*w*/*v*) ethanol (Grohmann et al. 1995; Table 4). However, these ethanol yields are difficult to compare due to differences in the biomass pretreatment and the fermentation conditions. *S. cerevisiae* fermentations were conducted with steam exploded citrus waste and an enzyme loading of 297 IU pectinase/g dry weight of citrus waste (dw). *E. coli* KO11 fermentations were conducted with citrus waste that was only enzymatically hydrolyzed, using 0.4 FPU cellulase/g dw cw, 12.4 IU pectinase/g dw cw, and 1.6 mg of β-glucosidase protein/g dw cw.

Better comparisons can be drawn from fermentations that were conducted with biomass that has had the same pretreatment. Grohmann et al. (1995) performed fermentations of citrus waste hydrolysate using *E. coli* KO11 and *S. cerevisiae*. The hydrolysate was formed by first grinding the citrus peel and then hydrolyzing the ground citrus waste with pectinase, cellulase, and β-glucosidase for 24 hours at 45 °C. Fermentation with *E. coli* KO11 increased ethanol titers by 25-35 % compared to *S. cerevisiae* fermentations (Grohmann et al. 1998).

Serial fermentations using *S. cerevisiae* and *E. coli* KO11 have been conducted to increase ethanol production from sugar beet pulp (Rorick et al. 2011). Serial addition of *E. coli* KO11 and then *S. cerevisiae* were unsuccessful due to the high concentrations of acetic acid produced by *E. coli* KO11. After fermentation with *E. coli* KO11 acetic acid levels reached 11 g/l, over twice the concentration *S. cerevisiae* can tolerate (Narendranath et al. 2001). Serial addition of *S. cerevisiae* followed by *E. coli* KO11 produced 2.97 % (*w*/*v*) ethanol, 0.37 % (*w*/*v*) more than fermentations conducted with *E. coli* KO11 alone (Table 5). However, maximum ethanol production was not reached until 216 h. *E. coli* KO11 only fermentations reached maximum ethanol concentrations much earlier (120 h). Therefore, *E. coli* KO11 only fermentations had higher volumetric productivity and essentially the same yield.

Peterson (2006) compared another strain of *E. coli*, strain LY01 to *S. cerevisiae* (Peterson 2006; Table 5) using sugar beet pulp as the substrate. Strain LY01 was isolated from *E. coli* KO11 and is more ethanol tolerant than KO11 (Yomano et al. 1998). Sugar beet pulp was autoclaved (121 °C/1 atm/20 min) and enzymatically hydrolyzed with 10.5 FPU cellulase/g dry weight of sugar beet pulp (dw sbp) and 240.8 IU pectinase/g dw sbp for 24 h at 42 °C and a starting pH of 5.0. LY01 produced 4 % (*w*/*v*) ethanol and *S. cerevisiae* produced 1.6 % (*w*/*v*) ethanol.

Recently, *E. coli* strains LY01 and KO11 have been engineered to further improve their ability to ferment pectin-rich biomass. One strain, *E. coli* LY40A, was engineered from *E. coli* KO11 by integrating the *casAB* operon from *Klebsiella oxytoca* into the *E. coli* genome (Edwards et al. 2011). The *casAB* operon encodes cellobiose phosphoenolpyruvate-dependent phosphotransferase genes which allows LY40A to uptake and metabolize cellobiose (Lai et al. 1997; Edwards et al. 2011). Sugar beet pulp fermentations conducted with LY40A produced 2.77 % (*w*/*v*) ethanol, while those conducted with *S. cerevisiae* only produced 1.74 % (*w*/*v*) ethanol. In an effort to further reduce the commercial enzyme load needed for pectin-rich biomass degradation, genes from *Erwinia chrysanthemi* were added to LY40A which allowed the organism, *E. coli* JP08C, to degrade pectin in sugar beet pulp to galacturonic acid monomers (Edwards et al. 2011). Pectate lyase E first hydrolyzed pectin into short chained oligogalacturonides. Oligogalacturonide lyase then degraded the oligogalacturonides into monomeric sugars which JP08C could ferment to ethanol. JP08C has been shown to increase ethanol yields in fermentations conducted with low commercial enzyme loadings, but the process has yet to be optimized for the production of industrially relevant levels of ethanol (Edwards et al. 2011).

## Fermenting with other ethanologens

Other organisms have been used for ethanol production from pectin-rich materials as well. Research has focused on strains that are thermotolerant, like the yeast *Kluyveromyces marxianus*, strains that can produce their own cell wall degrading enzymes, like the bacterium *E. chrysanthemi*, or organisms that are able to metabolize a wide variety of sugars, like the bacterium *K. oxytoca*. *K. marxianus* ferments hexose sugars to ethanol via a homoethanol pathway. Both bacterial ethanologens use the mixed acid fermentation pathway to metabolize sugars and will produce organic acid co-products similarly to *E. coli*.

The thermotolerance of *K. marxianus* is economically advantageous since the price of cooling fermentors could be reduced. Strains of *K. marxianus* isolated from sugar cane mills can produce ethanol at temperature as high as 47 °C (Anderson et al. 1986). A comparison of ethanol production from *K. marxianus* and *S. cerevisiae* in orange processing waste pretreated with steam expansion demonstrated that *K. marxianus* was capable of producing ethanol titers similar to that of *S. cerevisiae*. However, *K. marxianus* required a higher inoculation level than *S. cerevisiae* to produce comparable amounts of ethanol (Widmer et al. 2009). Further research is still required to understand the benefits of fermenting pectin-rich materials with *K. marxianus* instead of *S. cerevisiae*. Unfortunately, *K. marxianus*, like *S. cerevisiae*, is unable to naturally ferment pentose and acidic sugars.

Another option is the bacterium *E. chrysanthemi*, which can degrade plant cell wall components. Fermentations of dilute sulfuric acid and autoclaved pretreated citrus waste using *E. chrysanthemi* EC16 produced less ethanol than the fermentations conducted with *S. cerevisiae* (Wilkins et al. 2007b) and *E. coli* KO11 (Grohmann et al. 1995), described earlier (Table 4). *E. chrysanthemi* EC16 contains the PET operon from *Zymomonas mobilis* on the plasmid pLOI555 which increases the organisms ethanol production and decreases the final concentration of co-products (Beall and Ingram 1993). *E. chrysanthemi* EC16 only produced 1.28 % *w*/*v* ethanol (Grohmann et al. 1998).

Direct comparison of ethanol titers from biomass exposed to the same pretreatment gave similar results. *E. chrysanthemi* EC16 fermentations of sugar beet pulp produced 1.97 % (*w*/*v*) ethanol, less than the 2.55 % (*w*/*v*) ethanol produced by *E. coli* KO11 on the same substrate (Doran et al. 2000; Table 5). However, *E. chrysanthemi* EC16 was able to produce more ethanol than *E. coli* KO11 when no fungal enzymes were present, but these yields were low (Doran et al. 2000).

Doran et al. (2000) also conducted fermentations of sugar beet pulp with *K. oxytoca* P2, a strain with the PET operon chromosomally integrated (Wood and Ingram 1992). *K. oxytoca* is capable of fermenting a wide variety of pentose and hexose sugars including cellobiose, cellotriose, xylobiose, and xylotriose (Burchhardt and Ingram 1992; Wood and Ingram 1992). Like *E. chrysanthemi* EC16, when no commercial enzymes were added *K. oxytoca* P2 out produced *E. coli* KO11 but ethanol yields were low. In the presence of commercial enzymes *K. oxytoca* P2 produced 1.55 % (*w*/*v*) ethanol, lower than both *E. coli* KO11 and *E. chrysanthemi* EC16 ethanol production.

Some organisms combine high thermotolerance with cell wall degradation enzymes. These organisms have mainly been studied for their production of thermotolerant enzymes for food industries, less is known about their ethanol production from pectin-rich material. Fermentations conducted by Spinnler et al. (1986) of sugar beet pulp using *Clostridium thermocellum* demonstrated the organism’s propensity to produce acetate instead of ethanol when fermenting pectin-rich material. During the fermentation of 50 % (*w*/*v*) sugar beet pulp, 0.78 % (*w*/*v*) acetic acid was produced, while only 0.32 % (*w*/*v*) ethanol was produced (Table 5; Spinnler et al. 1986). It is unclear if the fermentations could reach industrially viable production levels if commercial enzymes were added to augment the activity provided by the organisms’ native cell wall degradation enzymes.

A third approach, bioengineering a homoethanol producing organism with narrow substrate utilization, *Z. mobilis*, to metabolize new sugars, has been considered but has yet to be tested on pectin-rich materials. *Z. mobilis* AX101 had been engineered to ferment xylose and arabinose along with glucose which is part of its native pathway (Mohagheghi et al. 2002). Fermentations of pure sugars have shown that AX101 is capable of metabolizing glucose, arabinose, and xylose into ethanol. However, these sugars were not used simultaneously and the organism does not ferment galacturonic acid.

## Concerns when fermenting pectin-rich materials

All of the ethanologens described above produce some acetate during pectin-rich biomass fermentations, except *S. cerevisiae*, *K. marxianus*, and *Z. mobilis* AX101 which are unable to metabolize galacturonic acid. The production of side products like acetate decreases the amount of ethanol that can be produced during fermentation. *E. coli* KO11, *E. chrysanthemi* EC 16, and *K. oxytoca* produced 0.23 (g/g), 0.38 (g/g), and 0.34 (g/g) acetate from 20 g/L fermentations of galacturonic acid, respectively (Doran et al. 2000). The metabolism of one mole of galacturonic acid produces one mole ethanol and one mole acetate due to the higher oxidation state of galacturonic acid in comparison to other sugars. Therefore, more molecules of NAD(P)H are required to ferment galacturonic acid, this is balanced by using the pyruvate formate lyase pathway which produces both ethanol and acetate (Grohmann et al. 1994b, 1995,1998).

Another concern when fermenting pectin-rich residues is citrus waste specific, the presence of the inhibitor D-limonene, an aromatic monoterpene that comprises about 86–95 % of the essential oils in citrus waste (Shaw 1979) and is present in citrus waste hydrolysate at concentrations of approximately 1.4 % (*v*/*v*; Grohmann et al. 1994a). Concentrations of peel oil between 0.05–0.1 % have been shown to have inhibitory effects on ethanol concentrations during the production of wine by yeast (von Loesecke 1934). Terpenes are believed to disrupt cellular membranes resulting in the release of cellular components, and the dissipation of the proton motive force and K^+^ gradient (Andrews et al. 1980; Uribe et al. 1985; Koroch and Juliani 2007).

Ethanol production from citrus waste by *S. cerevisiae* begins to decrease when D-limonene concentrations increase above 0.12 % (*v*/*w*; Wilkins et al. 2007b). The addition of peel oil, which contains D-limonene, to pure sugar or filtered citrus waste hydrolysate fermentations conducted with yeast (*S. cerevisiae* or *K. marxianus*) also decreased ethanol yields (Grohmann et al. 1994a; Wilkins et al. 2007a).

It has been suggested that gram-negative organisms, like *E. coli*, tend to be more resistant to some terpenes, including limonene, possibly due to the protection provided by their outer membrane (Andrews et al. 1980; Helander et al. 1998; Mann et al. 2000). Kim et al. (1995) observed the inhibitory effects of a variety of essential oil components on pathogenic bacteria. Limonene was shown to have no inhibitory effect against two strains of *E. coli*; however, it did inhibit growth of the gram-positive bacterium, *Listeria monocytogenes* (Kim et al. 1995). However, more recent studies have shown that limonene levels as low as 0.03 % (*v*/*v*) to be inhibitory to *E. coli* (Dunlop et al. 2011).

Fortunately, limonene can be removed from citrus waste by steam stripping the waste. Limonene is a valuable co-product of citrus waste fermentation and is generally recognized as safe (GRAS) by the Code of Federal Regulation. It is often used as a flavoring and fragrance, but has also been used as a solvent for cleaning supplies, a treatment for cholesterol containing gallstones, and a holistic treatment for gastroesophageal reflux disease and heartburn (Sun 2007). Removing limonene from the fermentation not only helps ethanol production but results in a more economically viable process. Zhou et al. estimated that limonene recovery could cut the cost of ethanol production from citrus waste by over $0.50/gal of ethanol (Zhou et al. 2007).

## Conclusions

Pectin-rich residues are generated as waste products from industrial processing of fruits and vegetables like apples, citrus, and sugar beets. In the USA, approximately 2.8 million tons (dw) of pectin-rich material is produced each year (Table 1). There are many options for disposal of this waste, from landfilling to the production of high-value products like aroma compounds. Currently most of this material is used for animal feed or put in landfills

Here we have reviewed another option, fermenting the biomass for fuel ethanol. Based on the amount of pectin-rich biomass produced annually in the USA, approximately 1 million tons or 335 million gallons of ethanol could be produced from these residues (Table 1). However, for this to be possible all of the sugars must be catabolized to ethanol. Of the possible ethanologens described above, *E. coli* is currently the best option for pectin-rich biomass fermentation. It can metabolize all of the sugars present in the biomass and has been engineered to produce high ethanol yields with limited unwanted co-products.
